# Phosphoenolpyruvate carboxykinase and the critical role of cataplerosis in the control of hepatic metabolism

**DOI:** 10.1186/1743-7075-2-33

**Published:** 2005-11-21

**Authors:** Parvin Hakimi, Mark T Johnson, Jianqi Yang, David F Lepage, Ronald A Conlon, Satish C Kalhan, Lea Reshef, Shirley M Tilghman, Richard W Hanson

**Affiliations:** 1 Department of Biochemistry, Case Western Reserve University School of Medicine, Cleveland, OH, USA; 2 Department of Pediatrics, Case Western Reserve University School of Medicine, Cleveland, OH, USA; 3 Department of Genetics, Case Western Reserve University School of Medicine, Cleveland, OH, USA; 4 Schwartz Center for Metabolism and Nutrition, MetroHealth Medical Center, Cleveland, OH, USA; 5 Department of Biochemistry, Hebrew University-Hadassah Medical School, Jerusalem, Israel; 6 Department of Molecular Biology, Princeton University, Princeton, NJ, USA

## Abstract

**Background:**

The metabolic function of PEPCK-C is not fully understood; deletion of the gene for the enzyme in mice provides an opportunity to fully assess its function.

**Methods:**

The gene for the cytosolic form of phosphoenolpyruvate carboxykinase (GTP) (EC 4.1.1.32) (PEPCK-C) was deleted in mice by homologous recombination (PEPCK-C^-/- ^mice) and the metabolic consequences assessed.

**Results:**

PEPCK-C^-/-^ mice became severely hypoglycemic by day two after birth and then died with profound hypoglycemia (12 mg/dl). The mice had milk in their stomachs at day two after birth and the administration of glucose raised the concentration of blood glucose in the mice but did not result in an increased survival. PEPCK-C^-/- ^mice have two to three times the hepatic triglyceride content as control littermates on the second day after birth. These mice also had an elevation of lactate (2.5 times), β-hydroxybutyrate (3 times) and triglyceride (50%) in their blood, as compared to control animals. On day two after birth, alanine, glycine, glutamine, glutamate, aspartate and asparagine were elevated in the blood of the PEPCK-C^-/- ^mice and the blood urea nitrogen concentration was increased by 2-fold. The rate of oxidation of [2-^14^C]-acetate, and [5-^14^C]-glutamate to ^14^CO_2 _by liver slices from PEPCK-C^-/- ^mice at two days of age was greatly reduced, as was the rate of fatty acid synthesis from acetate and glucose. As predicted by the lack of PEPCK-C, the concentration of malate in the livers of the PEPCK-C^-/- ^mice was 10 times that of controls.

**Conclusion:**

We conclude that PEPCK-C is required not only for gluconeogenesis and glyceroneogenesis but also for *cataplerosis *(i.e. the removal of citric acid cycle anions) and that the failure of this process in the livers of PEPCK-C^-/- ^mice results in a marked reduction in citric acid cycle flux and the shunting of hepatic lipid into triglyceride, resulting in a fatty liver.

## Background

The factors that regulate the transcription of the gene for the cytosolic form of phosphoenolpyruvate carboxykinase (GTP) (EC 4.1.1.32) (PEPCK-C) have been studied in great detail over the years [1]. However, its metabolic role in the various tissues in which the gene is expressed has received far less attention. Discussion of the function of PEPCK-C in mammals has largely been confined to its role in gluconeogenesis, with only a brief mention of alternative possibilities. PEPCK-C has become an important marker for hepatic gluconeogenesis; studies of the mechanisms involved in diabetes and related diseases, often include an analysis of the alterations in the mRNA for PEPCK-C. What is seldom mentioned, however, is the fact that PEPCK-C activity is present in a number of tissues that *do not *make glucose, including white and brown adipose tissue, mammary gland during lactation, small intestine, brain, lung, muscle and a number of others (see [2] for a review of this subject). Besides its role in *gluconeogenesis*, PEPCK-C is involved in *glyceroneogenesis *in adipose tissue [3] and liver [4,5]. This pathway is an abbreviated version of gluconeogenesis and results in the synthesis of glyceride-glycerol from precursors other than glucose or glycerol [6,7]. PEPCK-C can also catalyze the *formation *of oxalacetate, a citric acid cycle intermediate; it has been suggested that the enzyme "refills" the cycle during periods of biosynthesis (a process known as *anaplerosis*). However, the major biosynthetic tissues such as the liver and adipose tissue have considerable activity of pyruvate carboxylase, which itself generates oxalacetate. PEPCK-C is also the major *cataplerotic *enzyme, (cataplerotic enzymes remove citric acid cycle anions that are formed by the entry of the carbon skeletons of amino acids into the cycle). Both gluconeogenesis and glyceroneogenesis are cataplerotic processes, which use intermediates of the citric acid cycle for their specific biosynthetic process. As we will show in this paper, cataplerosis is a critical metabolic function. Finally, Hahn and Nowak [8] proposed that in brown adipose tissue a cycle operates between the mitochondria and the cytosol in which PEPCK-C converts GTP to GDP, to insure a continued citric acid cycle flux (GDP is a substrate for succinyl CoA synthase).

Ablating the gene for PEPCK-C in mice provides an opportunity to better assess the metabolic role of the enzyme in mammalian tissues. The gene for PEPCK-C has been deleted specifically in the livers of mice by She et al [9], who noted that the mice had a greatly reduced rate of hepatic gluconeogenesis from a variety of precursors. However, the animals did not develop hypoglycemia, even after as much as 48 hrs of fasting and could be induced to develop diabetes [10]. The absence of PEPCK-C in the liver also resulted in a decrease in the level of glycogen in both the liver and muscle; there was also diminished whole-body glucose turnover. Mice in which the gene for PEPCK-C has been totally deleted in all tissues die in the first two days after birth with profound hypoglycemia. These findings indicate that the kidney (the second gluconeogenic organ) can make sufficient glucose to provide for the needs of the animal during fasting and that it responds to the hormonal alterations characteristic of diabetes by increasing renal glucose output.

A surprising finding from the studies of She et al [9] was the observation that the ablation of the gene for PEPCK-C caused the mice to develop a fatty liver. As we will report here, the fatty liver appears as early as the end of the first day after birth and is accompanied by a 5-fold elevation in the concentration of triglyceride in the blood. While not expected by conventional metabolic logic, the accumulation of triglyceride in the liver could be the result of a failure of the development of glyceroneogenesis in adipose tissue or to a loss of gluconeogenesis (the major cataplerotic pathway in the liver) in the absence of PEPCK-C. In this paper we assess the impact of a loss of PEPCK-C in the perinatal period and evaluate the various functions suggested for this enzyme in energy metabolism.

## Methods

### Materials

Restriction enzymes, Taq DNA polymerase and proteinase K, NAD, NADH, lactate dehydrogenase, and malate dehydrogenase were purchased from Roche Applied Science (Indianapolis, IN). QuickPrep total RNA kit, [5-^14^C]-sodium glutamate, [U-^14^C]-glucose, and [2-^14^C]-sodium acetate was purchased from Amersham Biosciences Corp. (Piscataway, NJ). Triglyceride (GPO) Liquid Reagent Set was purchased from Pointe Scientific, Inc. (Lincoln Park, MI). The NEFA kit was from Wako Chemicals USA, Inc. (Richmond, VA). The embryonic stem cells used in this study were a generous gift from Janet Rossant, University of Toronto.

### Generation and maintenance of PEPCK-C^-/- ^mice

**Figure 1:**
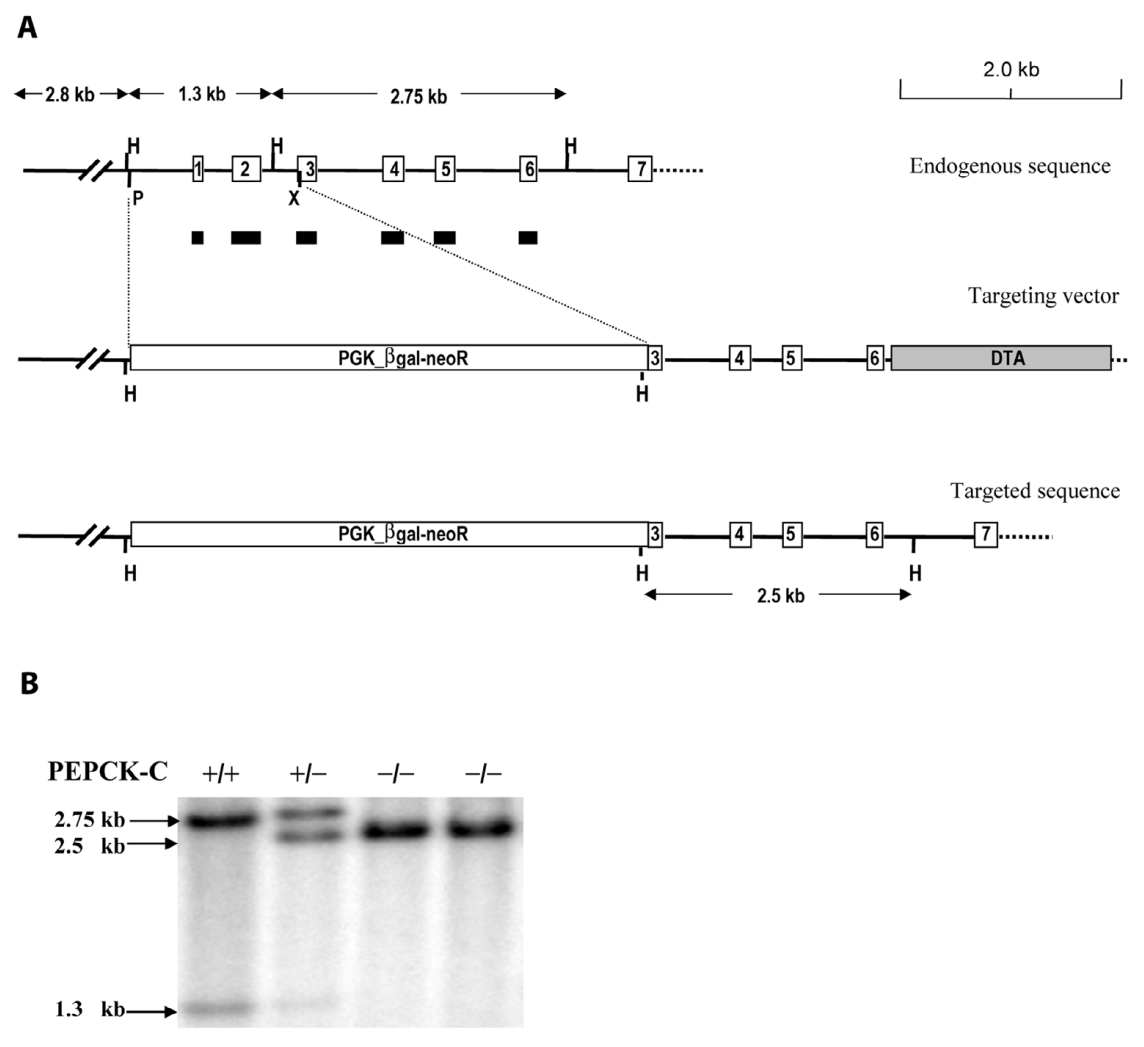
**Generation of PEPCK-C^-/- ^mice**. Panel A. Diagram of the targeting vector, which is aligned with endogenous sequence of PEPCK-C gene. The sequence between Pst I and Xho I, which covers part of the gene promoter and the exons 1 and 2 in endogenous gene, was replaced with Neo resistance gene (PGK_βgal-neoR) in the targeting vector. H, Hind III; P, Pst I; X, Xho I. PGK_βgal-neoR, the phosphoglycerate kinase gene promoter drives a fusion gene of βgal and neoR. DTA, the diphtheria toxin A chain. Panel B. Genotyping of the PEPCK-C^-/- ^mice by Southern blotting. Genomic DNA was digested with Hind III and hybridized with a PEPCK-C cDNA probe composed of exons 1~6 of the rat PEPCK-C gene (black bars). For the wild type allele of PEPCK-C, 1.3-kb and 2.75-kb fragments were detected; only one fragment (2.5 kb) was excepted for the targeted allele.

The gene for PEPCK-C was deleted in the mice as described in figure 1. A targeting vector was constructed in which the region of the PEPCK-C gene extending from a Pst I site at ~-300 bp in the PEPCK-C gene promoter to an Xho I site in exon three of the structural gene, was substituted with the PGK_βgal-neoR gene (figure 1A). The neomycin resistance gene (neo) and the diphtheria toxin A chain (DTA) were used as positive and negative selection markers, respectively. The targeting vector was linearized by Not I digestion and electroporated (25 μg of DNA) into E14 embryonic stem cells. Targeted clones that underwent homologous recombination were identified, using Southern blot hybridization of digested genomic DNA from ES cells. The probe used was a PEPCK-C cDNA sequence composed of exons one to six of the mouse PEPCK-C gene (figure 1A). Two out of eight hundred of the G418 resistant clones, that had been screened, showed correct homologous recombination. These clones were subsequently injected into C57BL/6 blastocytes to produce chimeric mice, which were then crossed with C57BL/6 mice to establish germ-line founders. The resulting offspring were genotyped by using Southern blotting or PCR.

### Metabolic studies

PEPCK-C^-/- ^mice and control littermates at one to three days of age were killed by decapitation. Their livers and blood were collected. Livers slices (5 to 10 mg) were prepared and incubated in a 10-ml Erlenmeyer flask, containing 1 ml of reaction buffer, which was composed of Krebs Ringer bicarbonate buffer at pH 7.4, 1 % defatted bovine serum albumin, and one of following substrates: 1 μCi [U-^14^C] glucose (5 mM), 1 μCi [2-^14^C] sodium acetate (2 mM), or 1 μCi [U-^14^C] sodium glutamate (1 mM). The flask had a thin rubber stopper that served both as a cap to close the flask and to hold a plastic bucket. The incubation was carried out at 37°C for 2 h in an atmosphere of O_2_/CO_2 _(95%/5%), in a shaking water bath. At the end of the incubation period, 200 μl of hyamine hydroxide was injected through the rubber stopper into the hanging bucket and 0.5 ml of 10% perchloric acid was injected into the incubation medium, to insure the complete liberation of CO_2_. After shaking for an addition 30 min, the tissue was removed, rinsed in saline and transferred to 2 ml of chloroform-methanol (2:1 v/v) for the extraction of total lipids. The lipid was extracted and its radioactivity determined. The hanging buckets containing the hyamine hydroxide were dissolved in liquid scintillation fluid and the radioactivity measured using a liquid scintillation spectrometer.

### Body composition

Mice were killed at two days of age and carcass analysis performed as described by Leischner et al.

### Histology of the Liver

Segments of livers from 2-day-old mice were fixed in 10% formalin (Sigma, St. Louis, MO) at 4°C. The tissues were embedded in paraffin and stained with hematoxylin and eosin by the Pathology Core Facility (Case Western Reserve University, Cleveland, OH). The photographs were taken at 400× magnification.

### DNA Analysis

DNA was isolated from the tails of the mice by lysis overnight at 55°C in a buffer containing 50 mM KCl, 10 mM Tris-HCl, pH 8.3, 2.5 mM MgCl_2_, 0.1% gelatin, 0.45% Nonidet P-40, 0.45% Tween20, and 24 mg/ml of proteinase K. The DNA was digested with Hind III, and the resulting fragments were separated by electrophoresis on 1% agarose gel, transferred to Gene Screen Plus^®^, and hybridized to a cDNA probe for PEPCK-C.

### RNA analysis

Total RNA was extracted from the livers of one and two-day old mice using a QuickPrep total RNA kit (Amersham Pharmacia Biotech) by a modified acid-phenol/guanidine thiocynate procedure [11] and Northern blotting was performed as described in detail previously [12]. Briefly, 20 μg of total RNA was separated by electrophoresis on an agarose gel, transferred to a Gene Screen Plus membrane and hybridized with a 1.0kb Sma I fragment of the 3'end of the PEPCK-C cDNA. The DNA probe was labeled by using [α-^32^P]-dCTP.

### Biochemical analysis

The following metabolites were measured in the blood of mice killed up to two days after birth. Blood glucose was determined using an Encore^® ^Glucometer. The concentrations of triglyceride, β-hydroxybutyrate, and urea nitrogen in the blood were determined by Veterinary Diagnostic Services in Marshfield Laboratories (Marshfield, WI), using standard clinical procedures. The concentrations of amino acids in plasma were measured with a high-pressure liquid spectrophotometer equipped with a fluorescent detector, using a *o*-phthaldehyde derivative and pre-column derivatization [13]. Livers of mice were used to determine the content of glycogen [14] or were freeze-clamped and metabolites extracted as described previously [15]. Spectrophotometric methods were used to determine the concentrations of malate [16], lactate [17], pyruvate [18] and ammonia [19] in the liver extracts or in the blood. The concentration of hepatic triglyceride was determined using the triglyceride reagent set from Pointe Scientific, Inc. (Lincoln Park MI). Briefly, segments of liver were saponified in ethanol-KOH, the sample diluted 1:5 and one ml of triglyceride reagent added to 10 μl of diluted sample and incubated at 37°C for 5 min. The reaction product was analyzed using spectrophotometer (A_500_) and the concentration determined by linear regression using standards treated with one ml of triglyceride reagent. The activity of PEPCK-C in the liver cytosol of two-day-old PEPCK-C^-/- ^mice and control littermates was determined as described by Ballard and Hanson [20] and the activity expressed as units/g tissue, where one unit equals one μmole of substrate converted to product per min.

### Statistical analysis

All data are reported as means ± the SE. The statistical analysis was performed using SigmaPlot from Systat Software, Inc. (Point Richmond, CA).

## Results

**Figure 2:**
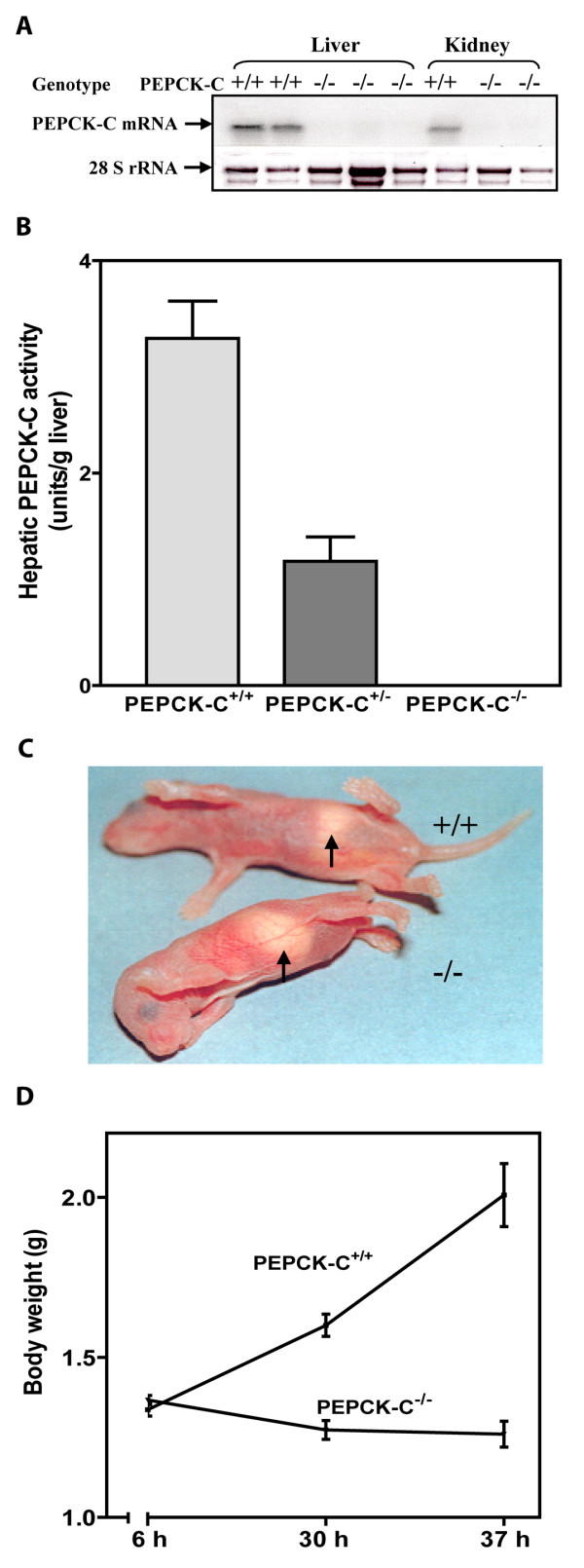
**Characterization of the PEPCK-C^-/- ^mice**. Panel A. A representative Northern blot for mRNA isolated from the liver and kidney of two-day-old mice. Panel B. PEPCK-C activity was determined in the livers of two-day-old mice. The results are expressed as the mean ± S.E. for three animals in each group. Panel C. Photograph of two-day-old PEPCK-C^-/- ^mice. Panel D. Growth retardation of PEPCK-C^-/- ^mice. Body weights of neonates were measure at 6, 30, and 37 h after birth. Three wild type mice and three PEPCK-C^-/- ^mice were used.

A line of mice was created (PEPCK-C^-/- ^mice) in which the gene for PEPCK-C was deleted by inserting the neo resistance gene, driven by the phosphoglycerate kinase gene promoter, into the PEPCK-C gene, thereby deleting a region of the gene from the Pst I site at -569 of the gene promoter to the XhoI site within the third exon (figure 1). The PEPCK-C^-/- ^mice had no detectable mRNA for PEPCK-C in the liver and kidney (the only tissues tested) (figure 2A) and no activity for the enzyme was noted in their livers (figure 2B). PEPCK-C^-/- ^mice were present in litters at the expected Mendalian ratio at birth, however, all of the PEPCK-C^-/- ^mice died within two to three days after birth. The animals had no distinguishing phenotype at birth, suckled normally and had milk in their stomachs (figure 2C). However, by one day after birth PEPCK-C^-/- ^the mice failed to gain weight (figure 2D) and had about half the total body fat content of control littermates (Table 1). Mice *heterozygous *for the PEPCK-C gene deletion (PEPCK-C^+/- ^mice) had only half the activity of the enzyme in their livers (figure 2B) and lived normally, with no observable metabolic abnormalities.

**Table 1:** Metabolic alterations in PEPCK-C^-/- ^mice at two days of age.

**Body composition**	**PEPCK-C^+/+^**	**(n)**	**PEPCK-C^-/-^**	**(n)**
**% Water**	80.3 ± 0.30	3	79.26 ± 0.22	3
**% Fat**	3.48 ± 0.36	3	1.85 ± 0.57	3
**% Lean Mass**	16.42 ± 0.63	3	18.88 ± 0.78	3

**Metabolites in liver**				

**Triglyceride (μmoles/g)**	4.17 ± 1.00	5	9.58 ± 1.75	5
**Lactate (μmoles/g)**	0.19 ± 0.02	3	0.37 ± 0.09	3
**Malate (μmoles/g)**	0.24 ± 0.05	3	2.84 ± 1.58	3

**Metabolites in plasma**				

**Triglyceride (mg/dl)**	23.7 ± 6.3	3	36.3 ± 21.5	2
**Ammonia (μmoles/ml)**	0.14 ± 0.02	5	0.45 ± 0.28	4
**BUN (mg/dl)**	29.0 ± 7.0	2	65.5 ± 28.5	2
**Lactate (μmoles/ml)**	0.28 ± 0.13	3	0.72 ± 0.47	3
**β-Hydoxybutyrate (μmoles/ml)**	5.83 ± 0.21	3	17.95 ± 0.70	3

The values are expressed as the mean ± S.E. for the number of animals shown in parenthesis

**Figure 3:**
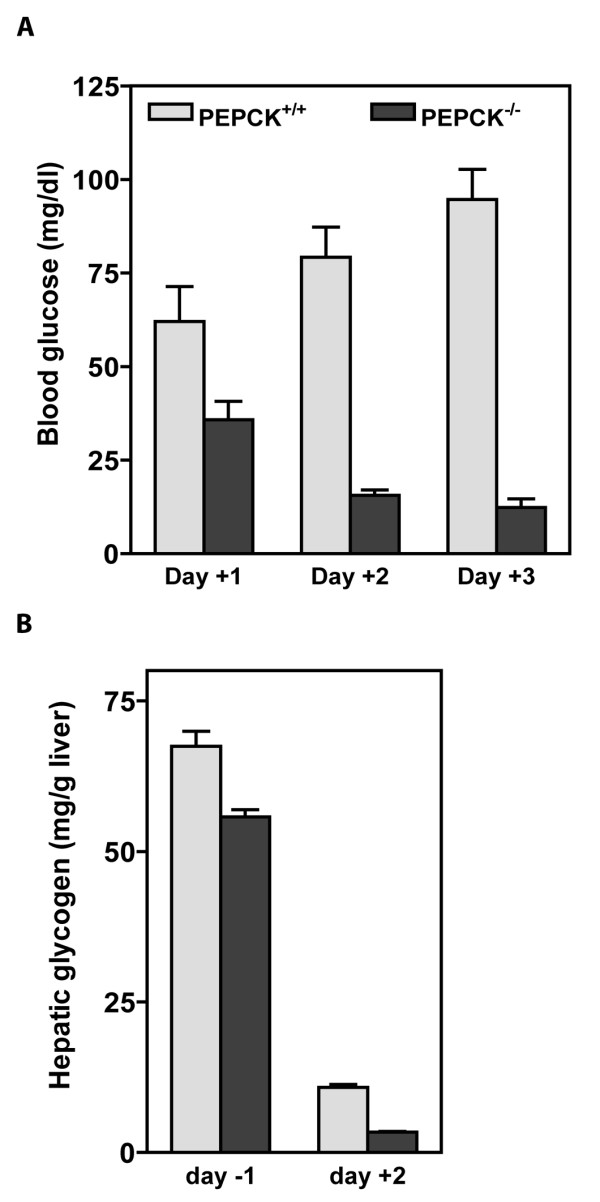
**Alterations in glucose homeostaisis in PEPCK-C^-/- ^and control mice during the perinatal period**. Panel A Hypoglycemia in PEPCK-C^-/- ^mice. The concentration of blood glucose was measured in mice at one, two and three days after birth. The results are expressed as the mean ± S.E. for from three to six animals. Panel B. Increased mobilization of hepatic glycogen. The hepatic glycogen level was analyzed at the age of fetal day 19 (day -1) and neonatal day (day +2). The results are expressed as the mean ± S.E. for three animals in each group.

The effect of a deletion of the gene for PEPCK-C on glucose homeostasis is evident from the changes in the concentration of glucose in the blood of PEPCK-C^-/- ^mice, as compared to control littermates. PEPCK-C activity first appears in the liver at birth [20]. Thus, a deletion of the gene for PEPCK-C should not have a significant effect on glucose homeostasis before birth, since the glucose needed for fetal development is provided by the mother. One day after birth the levels of glucose in the blood of PEPCK-C^-/- ^mice was only 60% of that in controls, and by day three the concentration of glucose in the blood of these mice was 12.3 mg/dl (figure 3A). It is likely that a failure of gluconeogenesis, caused by the ablation of PEPCK-C [10] contributed to the low level of glucose in the blood. The concentration of hepatic glycogen was determined at fetal day 19 (one day before birth) and at day two after birth in both PEPCK-C^-/- ^mice and controls (figure 3B). There was 10% less glycogen in the livers of PEPCK-C^-/- ^mice delivered one day before birth, as compared to control animals. By two days after birth the concentration of hepatic glycogen was markedly decreased in both PEPCK-C^-/- ^mice and controls, but the overall level of glycogen mobilization was much greater in the PEPCK-C^-/- ^mice (figure 3B). This is likely due to the need of he animal to rely the mobilized hepatic glycogen as a source of glucose for fuel metabolism. Interestingly, the administration of glucose by intraperitoneal injection did not rescue the mice from death at day two after birth (data not shown), suggesting that factors other than a disruption of glucose homeostasis were responsible for the death of the mice (i.e. the great accumulation of hepatic triglyceride or a failure of cataplerosis).

**Figure 4:**
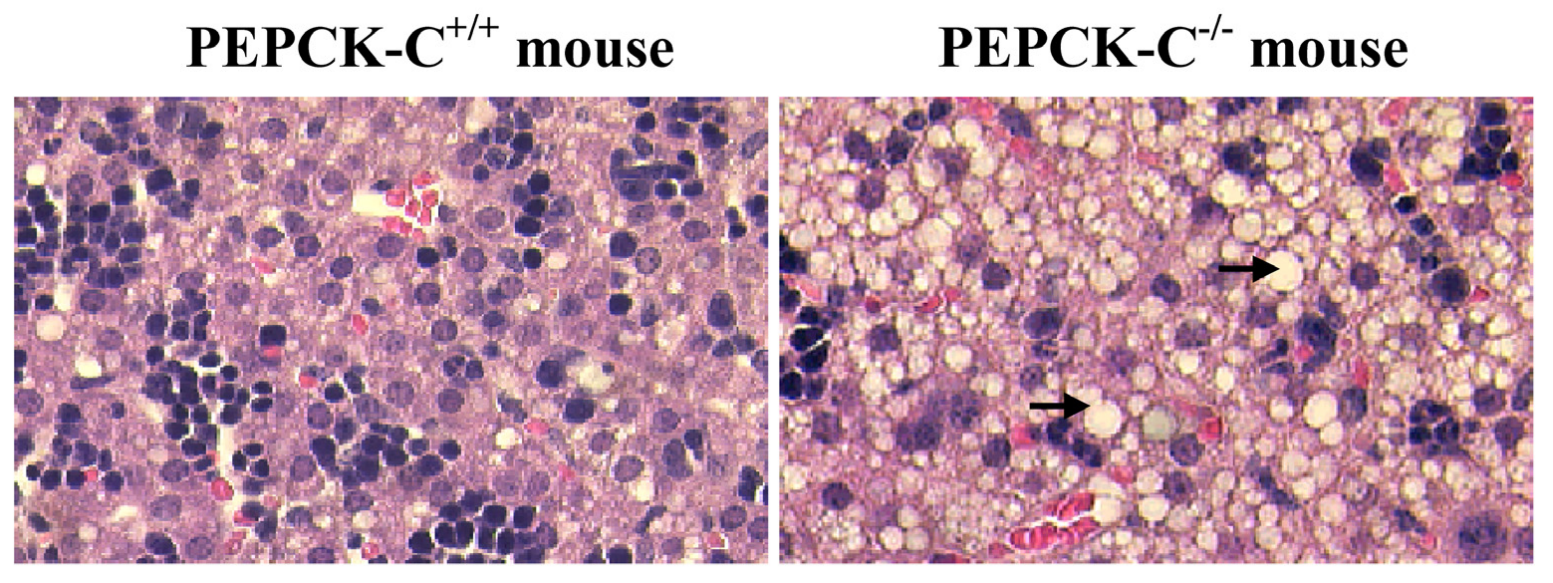
**Morphology of the livers of PEPCK-C^-/- ^and control mice**. Livers of two-day-old pups were analyzed with H&E staining. The arrow indicates fat accumulation in the liver of PEPCK-C^-/- ^mice.

Despite having less body fat, PEPCK-C^-/- ^mice had a marked hepatic lipid accumulation, which was visible when the liver was removed from the animal for biochemistry analysis. Histological analysis of the livers of animals at two days of age indicated extensive lipid infiltration of the liver (figure 4). This accumulation of lipid progressed until the mice died between two and three days after birth. The metabolic profile of the PEPCK-C^-/- ^mice at day two after birth is shown in Table 1. The concentration of triglyceride in the livers of these mice was twice that of controls; this was accompanied by a similar, almost 2-fold difference in the level of triglyceride in the blood of the PEPCK-C^-/- ^mice. The concentration of β-hydroxybutyrate in the blood of PEPCK-C^-/- ^mice was three times that of control mice. In addition, the concentration of ammonia in the blood of the PEPCK-C^-/- ^mice was elevated 3-fold over that noted in the blood of control mice, while blood urea nitrogen (BUN) was increased by 2-fold (Table 1), suggesting an increased generation of amino nitrogen from the breakdown of amino acids.

Since the deletion of the gene for PEPCK-C should alter the levels of intermediates in related metabolic pathways, we determined the level of malate and lactate in the liver by freeze-clamping the livers of PEPCK-C^-/- ^mice and controls at two days after birth. Intermediates were extracted from the frozen livers and their concentrations measured (Table 1). The concentration of malate was increased by 10-fold in the livers of PEPCK-C^-/- ^mice and the lactate concentration was elevated 2.5-fold. We did not distinguish between the malate in the cytosol and the mitochondria, but it is likely that there is an increase in the concentration of this citric acid cycle intermediate in both compartments of the hepatocyte. This finding of an elevated concentration of malate in the liver suggests that in absence of PEPCK-C the rate of oxalacetate conversion to PEP is limited and this intermediate is converted instead to malate, which accumulates. A similar increase in the concentration of malate in the livers of PEPCK-C^-/- ^mice was reported by She et al. [9].

It is not intuitive that the ablation of PEPCK-C, which is normally classified as a gluconeogenic enzyme, would result in profound fatty livers in the mice. However, PEPCK-C is a major cataplerotic enzyme, so that its absence in the liver would be predicted to alter the rate of citric acid cycle flux, since a major route for the disposal of carbon skeletons from the break down of amino acids would be blocked. This is demonstrated in experiments in which the rate of oxidation of acetate, glucose and glutamate to CO_2 _and their conversion to total lipid by liver slices from PEPCK-C^-/- ^mice was compared with control animals. The generation of ^14^CO_2 _from [2-^14^C]-acetate was 30% of the rate of oxidation of this compound by liver slices from control mice and the rate of hepatic lipid synthesis by PEPCK-C^-/- ^mice was negligible (Table 2). The same pattern was noted for the metabolism of [U-^14^C]-glucose. We also determined the rate of oxidation of [5-^14^C]-glutamate, which enters the citric acid cycle as α-ketoglutarate and must leave as malate or some other four-carbon anion to insure continuing carbon flux through the cycle. Gluconeogenesis is the most important route of disposal of the carbon skeletons that enter the citric acid cycle; in the absence of PEPCK-C the rate of oxidation of [5-^14^C]-glutamate to ^14^CO_2 _was markedly diminished (Table 2).

**Table 2:** The conversion of glucose, acetate and glutamate to CO_2 _and lipid by liver slices from PEPCK-C^-/- ^and control mice.

	**CO_2_**				**Total Lipid**			
**Substrates**	**PEPCK-C^+/+^**	(n)	**PEPCK-C^-/-^**	(n)	**PEPCK-C^+/+^**	(n)	**PEPCK-C^-/-^**	(n)
**Glucose-U-^14^C**	0.92 ± 0.26	(8)	0.30 ± 0.04	(5)	0.153 ± 0.009	(3)	0.063 ± 0.002	(2)
**Acetate-2-^14^C**	12.36 ± 0.88	(4)	2.72 ± 0.20	(5)	0.097 ± 0.018	(7)	0.002 ± 0.000	(2)
**Glutamate-5-^14^C**	5.15 ± 0.65	(2)	1.75 ± 0.65	(2)	1.778 ± 0.091	(4)	0.983 ± 0.098	(3)

The values are expressed as μmoles of substrate converted to product/hour/g liver and represent the mean ± SE of the mean for the number of determinations shown in parenthesis. Livers slices from two-day-old mice were incubated for 2 h in Krebs Ringer bicarbonate buffer, pH 7.4, containing substrates at the concentrations outlined in the Methods, and the rates of ^14^CO_2 _production and ^14^C incorporation into total lipid determined. The radioactivity was quantified using a liquid scintillation spectrometer.

**Figure 5:**
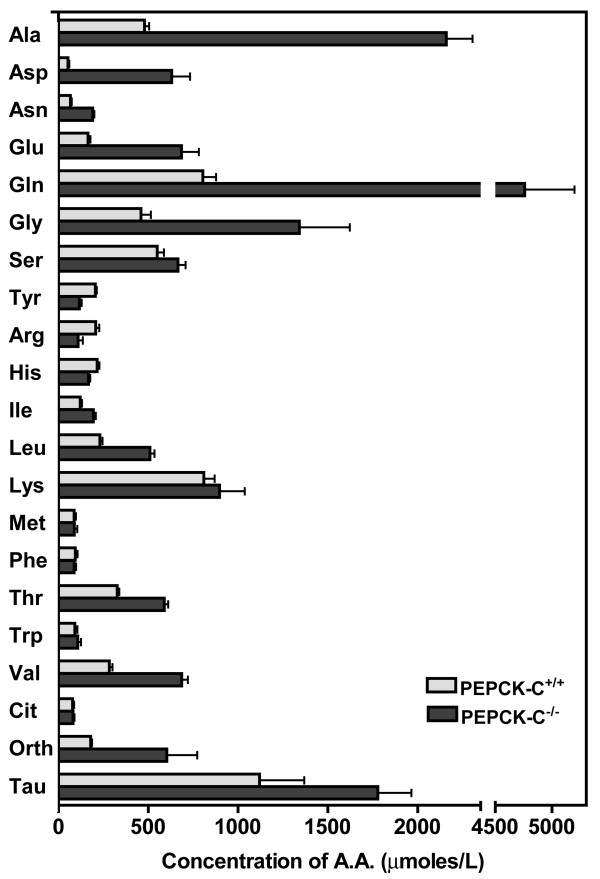
**The concentration of amino acids in the blood of PEPCK-C^-/- ^mice and controls**. The levels of amino acids were determined in mice at two days after birth. The results are expressed as the mean ± S.E. of three controls and four PEPCK-C^-/- ^mice.

The synthesis of non-essential amino acids from citric acid cycle intermediates, such as oxalacetate (from aspartate and asparagine) and α-ketoglutarate (from glutamate and glutamine) is potentially capable of relieving the accumulation of intermediates in the cycle that occurs in the livers of PEPCK-C^-/- ^mice [21]. We have measured the level of amino acids in the blood of PEPCK-C^-/- ^mice and controls (figure 5). At two days after birth, there was a marked increase in a number of the non-essential amino acids in the blood of PEPCK-C^-/- ^mice. The levels of alanine, aspartate, asparagine, glutamate, glutamine and glycine in the blood of the PEPCK-C^-/- ^mice were two to eight-fold higher than noted in control animals. This suggests that the carbon skeletons of these amino acids, which would normally enter the citric acid cycle, are prevented from being readily metabolized and the amino acids accumulate in the blood. It is also likely that some fraction of the non-essential amino acids were synthesized *de novo *from citric acid cycle intermediates, which accumulate in the liver due to the absence of PEPCK-C. This suggestion is supported by the increased concentration of ammonia in the blood of the PEPCK-C^-/- ^mice (see Table 1), which favors the formation of non-essential amino acids in the liver, especially glutamine, which was elevated five times over that noted in the blood of control animals. The concentration in the blood of a number of the essential amino acids, such as lysine, methionine, phenylalanine and tryptophan was not altered by ablation of PEPCK-C, while the levels of leucine, isolucine and valine were increased. The elevation in the level of ornithine, together with the elevated levels of both ammonia and urea in the blood of the PEPCK-C^-/- ^mice suggests elevated urea cycle flux to dispose of amino nitrogen from the breakdown of amino acids.

## Discussion

It is clear from this and other studies [9,10] that PEPCK-C is critical for life; its absence in the mouse results in death within the first two to three days after birth. We are aware of only one reported example of PEPCK-C deficiency in humans. In 1976, Vidnes and Sovik [22] reported the absence of PEPCK-C in the liver of an infant, which had persistent hypoglycemia and an early death, despite the presence of PEPCK-M. The rarity of PEPCK-C deficiency in humans further supports the physiological significance of this enzyme. The developmental profile of hepatic PEPCK-C is of interest in this regard. PEPCK-C is absent in the liver of all mammals during fetal development and appears dramatically at birth [20]. In contrast, the enzyme is present in the kidney [23] and may appear in other tissues before birth, but the exact pattern of development in tissues other than the kidney and liver has not been determined. As an example, the time course of development of PEPCK-C in white adipose tissue is currently unknown, despite the important role that PEPCK-C plays in this tissue [3,7]. Considerable PEPCK-M activity is present in mammalian liver before birth [24] but it is the appearance of PEPCK-C at birth and the marked alteration in the hepatic redox state, which occurs during this period [25], that are generally considered to be the important events in the initiation of hepatic gluconeogenesis. Since the role of PEPCK-C in gluconeogenesis is well established in all species [26], it is reasonable to assume that one of the causes of the death of the PEPCK-C^-/- ^mice is the profound hypoglycemia which occurs at two to three days after birth. However, providing glucose to the mice by intraperitoneal injection did not rescue the animals.

Mice with a *liver-specific *deletion of the gene for PEPCK-C survive the perinatal period, maintain normal glucose homeostasis during fasting and can be made diabetic [10]. Whole body glucose turnover in these mice is only slightly decreased, when compared to control animals. This suggests that the kidney can assume the function of the liver and make enough glucose for the needs of the animal. In addition, the liver can synthesize glucose from glycerol, a process that by-passes the reactions in the pathway of gluconeogenesis that require PEPCK-C. Since only 5–10% of the total PEPCK is mitochondrial in rodent species [27], it is unlikely that PEPCK-M plays a significant role in hepatic gluconeogenesis in mice lacking hepatic PEPCK-C. Mice lacking hepatic PEPCK-C have lower levels of liver glycogen two days after birth. As noted in Table 1, newborn PEPCK-C^-/- ^mice have considerably less glycogen than control littermates, presumably due to the need to mobilized glycogen to support the blood glucose levels in the absence of hepatic gluconeogenesis. However, She et al [9] reported that mice lacking PEPCK-C in the liver had a greatly reduced rate of glycogen synthesis in both the liver and the muscle.

The marked accumulation of fat in the livers of the PEPCK-C^-/- ^mice strongly suggests that there is another critical metabolic role for the enzyme (other than gluconeogenesis) which, when missing, can markedly alter the metabolic capacity of the animal. The concentration of hepatic triglyceride in mice two days after birth is twice that of control animals. The biochemical mechanisms responsible for the accumulation of triglyceride in PEPCK-C^-/- ^mice are complex. In the present paper, we present evidence that in the absence of PEPCK-C hepatic cataplerosis is greatly reduced, resulting in a reduction of the ability of the liver to appropriately oxidize acetyl CoA to CO_2 _in the citric acid cycle. This causes an increase in the synthesis of ketone bodies and a build up of fatty acids in the liver, with the subsequent synthesis of triglyceride. *Cataplerosis *describes reactions involved in the disposal of citric acid cycle intermediates generated by the entry of compounds into the cycle during the breakdown of amino acids and other metabolites (i.e. propionyl CoA), or by the carboxylation of pyruvate to oxalacetate [28]. The biological necessity for cataplerosis resides in citric acid cycle dynamics. The cycle oxidizes acetyl CoA to CO_2 _but cannot fully oxidize four or five carbon intermediates. This requires a series of reactions that are capable of efficiently removing citric acid cycle anions before they accumulate in the mitochondria. It is now recognized that PEPCK-C functions in cataplerosis in many tissue, not just in the more intensively studied tissues, such as the liver and kidney. For example, the net generation of alanine from glutamine, as occurs in the small intestine, involves PEPCK-C and cataplerosis. Glutamine carbon enters the citric acid cycle as α-ketoglutarate, is converted in the cycle to malate, which leaves the mitochondria, is oxidized to oxalacetate and then decarboxylated to PEP by PEPCK-C. Pyruvate kinase then converts the PEP to pyruvate, the pyruvate is transaminated by alanine aminotransferase to alanine, which is then released into the blood and transported to the liver. It has been estimated that as much as 40% of the alanine used for gluconeogenesis by the human liver during starvation is derived from the glutamine in the small intestine [29]. Alternatively, the pyruvate can be converted to acetyl CoA in the mitochondria for the generation of energy via the citric acid cycle. A similar role for PEPCK-C has been proposed for the conversion of other amino acids to alanine in the muscle [30]. Deleting the gene for PEPCK-C in tissues of the mouse, where it is normally present, will have the effect of altering cataplerosis and profoundly interfering with energy metabolism.

**Figure 6:**
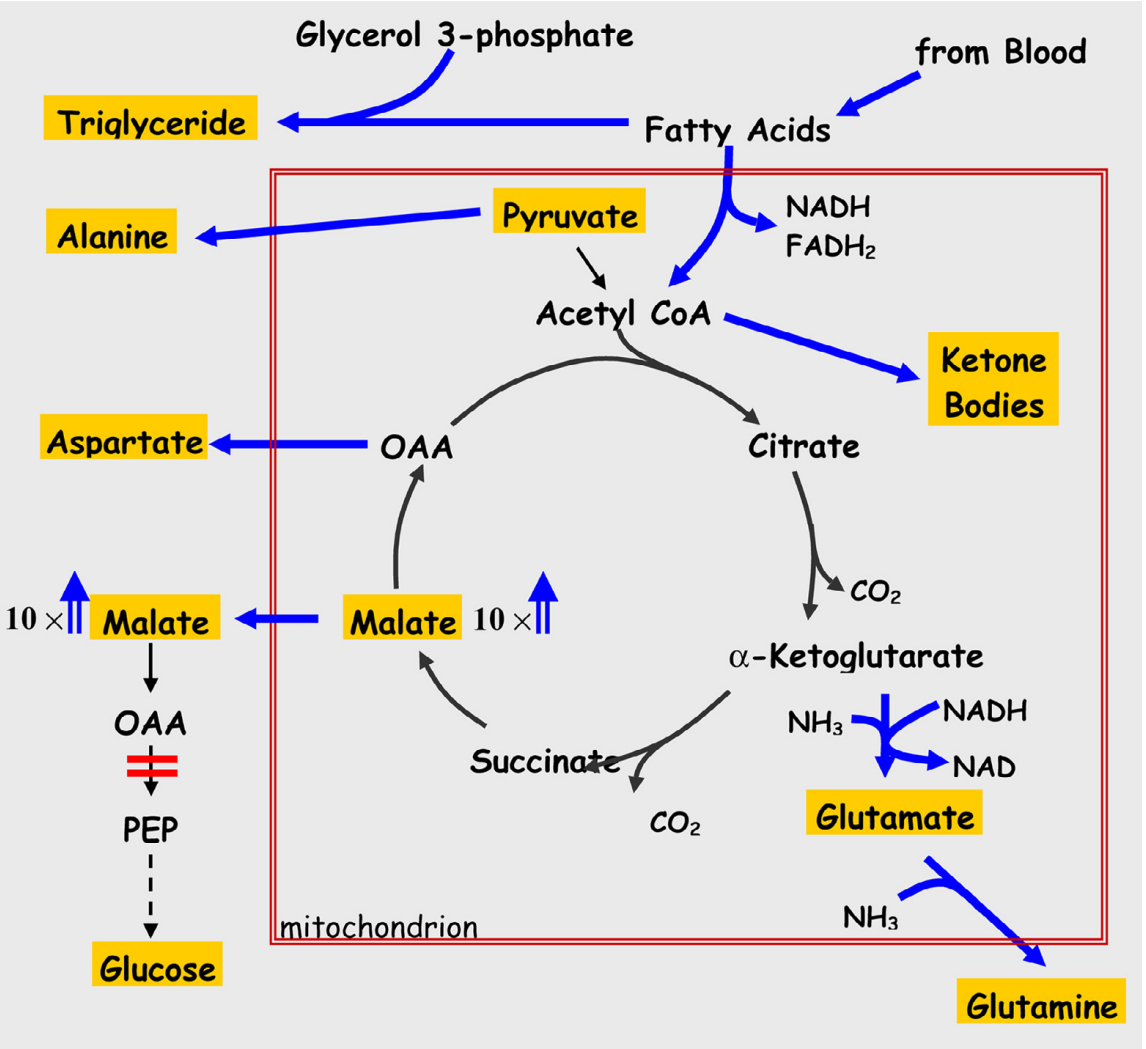
**The role of PEPCK-C in cataplerosis in the liver**. The reactions of the citric acid cycle are presented, with the major end-products shown in gold boxes; the large red box represents the mitochondrial membrane. The concentrations of amino acids, β-hydroxybutyrate, glucose and triglyceride were determined from blood samples, while the levels of malate and pyruvate were determined from freeze-clamped liver. The ablation of PEPCK-C (shown by a red bar) results in a 10-fold increase in the concentration of malate in the liver (we did not distinguish between malate in the cytosol and the mitochondria) and a build-up of cycle intermediates [23]. This leads to a decrease in the rate of citric acid cycle flux and the resultant accumulation of acetyl CoA, which is subsequently converted to ketone bodies and released by the liver. The rate of fatty acid oxidation in the PEPCK-C^-/- ^mice is also markedly decreased, resulting in an increase in triglyceride synthesis from these fatty acids that leads to the development of a fatty liver. There is also a marked increase in the concentration of amino acids in the blood that were generated from citric acid cycle intermediates. The increased rate of flux of intermediates leaving the citric acid cycle is denoted by heavy arrows.

The results of the present study suggest that in the absence of PEPCK-C in the liver the rate of removal of intermediates from the citric acid cycle is greatly reduced. This is illustrated in figure 6. The high concentration of malate (10 times that noted in controls) in the livers of PEPCK-C^-/- ^mice is most likely due to the lack of removal of oxalacetate in the absence of PEPCK-C. As malate accumulates, the rate of citric acid cycle flux is markedly reduced, resulting in a decrease in both the oxidation of acetyl CoA to CO_2 _and its conversion to fatty acid in the livers of PEPCK-C^-/- ^mice, as well as a reduction in the rate of oxidation of glucose and glutamate to CO_2 _(see Table 2). The acetyl CoA not utilized in the citric acid cycle is converted to ketone bodies, which increase in the blood of the PEPCK-C^-/- ^mice (Table 1). The decreased oxidation of acetyl CoA in the citric acid cycle results in the shunting of fatty acids into the synthesis of triglyceride, with the subsequent accumulation of fat in the liver. As would be predicted, the absence of PEPCK-C results in an increase in the level of a number of non-essential amino acids, including aspartate, asparagine, glutamate, glycine, alanine and glutamine that are synthesized from citric acid cycle anions. When hepatic PEPCK-C is ablated, the synthesis and release of these amino acids from the liver serves as a major cataplerotic process. An alteration in citric acid cycle flux would thus contribute to the observed accumulation of these amino acids in the blood. Clearly, the failure of the liver to carry out cataplerosis has far reaching effects on energy metabolism in the animal. It should be noted, however, that genetic disorders in humans in which the activity of pyruvate carboxylase is reduced results in the development of a fatty liver, suggesting that a failure of *anaplerosis *can also result in hepatic fat accumulation.

Mice with a liver-specific ablation of PEPCK-C have been used by Burgress et al [21] to determine the importance of this enzyme in citric acid cycle flux in the intact animal. They noted that after a 24 h fast virtually all of the newly synthesized glucose was from glycerol and the formation of glucose from citric acid cycle intermediates was negligible. Flux through the citric acid cycle was 10 to 40-fold lower than noted in the livers of control mice, which correlated with an accumulation of citric acid cycle intermediates. They concluded that in the absence of PEPCK-C there was a failure of cataplerosis, leading to a decreased citric acid cycle flux, decreased fatty acid oxidation and an accumulation of liver lipid. This finding is in accord with that described in this paper using mice in which the gene for PEPCK-C is deleted in all tissues and underscores the key role of PEPCK-C in the normal functioning of the citric acid cycle in mammals.

In addition to altering cataplerosis, the deletion of the gene for PEPCK-C will result in a decrease in glyceroneogenesis in tissues of the mouse. At birth the mouse ingests milk that is relatively high in fat (13%) and low in carbohydrate (3%) when compared with some other mammalian species (i.e. human). This dietary fat, while a good source of energy, is deposited in white adipose tissue during the early perinatal period. The relatively low level of carbohydrate in the diet suggests that glyceroneogenesis in *adipose tissue *could play an important role in controlling the rate of triglyceride deposition in this tissue during suckling. Studies in our laboratory, using *in vivo *tracer methodology, have shown that glyceroneogenesis in adipose tissue of the rat is the predominant source of glyceride-glycerol, even when the animals were fed a diet high in sucrose (Nye, Hanson and Kalhan, unpublished data). If glyceroneogenesis is playing an important role in the deposition of triglyceride in the adipose tissue of the newborn mouse, the absence of PEPCK-C in the tissue could explain the very high level of triglyceride accumulation noted in the livers of the PEPCK-C^-/- ^mice, since fatty acids not re-esterifed to triglyceride in adipose tissue, could end up being converted to triglyceride in the liver. In support of this suggestion, the tissue-specific ablation of PEPCK-C gene expression in adipose tissue of mice resulted in the development of a fatty liver.

## Conclusion

The studies presented here and the recent work on mice with a liver-specific deletion in the gene for PEPCK-C [9,10,21], emphasize the important role played by this enzyme in a number of metabolic process other than gluconeogenesis. It is likely that PEPCK-C is critical for cataplerosis, especially in those tissues in which significant levels of biosynthesis occur. The dynamic nature of the citric acid cycle and its role in both energy generation and biosynthetic process requires the coordinated activity of PEPCK-C to insure a balance between carbon input into the cycle and carbon outflow. The fact that this critical function of PEPCK-C has not been appreciated for so long, underlines the importance of genetically modified animal models as tools for better understanding the control of metabolism. As an example, the metabolic function of PEPCK-M has been virtually ignored over the years despite the fact that it represents 50% of the activity of PEPCK in humans. This neglect is ironic, since Utter and Kurahashi discovered PEPCK-M in the livers of chickens [31] and used it to delineate the pathway of gluconeogenesis. Our understanding of the biology of PEPCK will not be complete until we directly determine the metabolic role of PEPCK-M in mammalian species. In this regard, the major limiting factor is the fact that the animal species most easily genetically modified (the mouse) has only a marginal activity of PEPCK-M in any tissue. It should be possible, however, to introduce a transgene containing the gene PEPCK-M into tissues of PEPCK-C^-/- ^mice. This would allow us to assess the metabolic function of PEPCK-M in the absence of PEPCK-C; these studies are in progress.

## Competing interests

The author(s) declare that they have no competing interests.

## Authors' contributions

PH, made genetically modified mice and carried out molecular studies; MJ, DL and DAC provided guidance in aspects of the preparation of genetically modified of the mice; JY, assisted in the preparation and analysis of the data for the manuscript; SCK, provided amino acid analysis and data interpretation and helped in the preparation of the manuscript; SMT, participated in the development of the genetically modified mice; LR, participated in the experimental design and helped in the preparation of the manuscript; RWH, conceived of the study and worked on the experimental design, guided aspects of the work and wrote the manuscript.

## References

[B1] Chakravarty K, Cassuto H, Reshef L, Hanson RW (2005). Factors that control the tissue-specific transcription of the gene for phosphoenolpyruvate carboxykinase-C. Crit Rev Biochem Mol Biol.

[B2] Hanson RW, Patel YM, Meister A (1994). P-enolpyruvate carboxykinase: the gene and the enzyme. Advances in Enzymology.

[B3] Reshef L, Hanson RW, Ballard FJ (1970). A possible physiological role for glyceroneogenesis in rat adipose tissue. J Biol Chem.

[B4] Botion LM, Brito MN, Brito NA, Brito SR, Kettelhut IC, Migliorini RH (1998). Glucose contribution to in vivo synthesis of glyceride-glycerol and fatty acids in rats adapted to a high-protein, carbohydrate-free diet. Metabolism.

[B5] Kalhan SC, Mahajan S, Burkett E, Reshef L, Hanson RW (2001). Glyceroneogenesis and the source of glycerol for hepatic triacylglycerol synthesis in humans. J Biol Chem.

[B6] Reshef L, Olswang Y, Cassuto H, Blum B, Croniger CM, Kalhan SC, Tilghman SM, Hanson RW (2003). Glyceroneogenesis and the triglyceride/fatty acid cycle. J Biol Chem.

[B7] Hanson RW, Reshef L (2003). Glyceroneogenesis revisited. Biochimie.

[B8] Hahn P, Novak M (1975). Development of brown and white adipose tissue. J Lipid Res.

[B9] She P, Shiota M, Shelton KD, Chalkley R, Postic C, Magnuson MA (2000). Phosphoenolpyruvate carboxykinase is necessary for the integration of hepatic energy metabolism. Mol Cell Biol.

[B10] She P, Burgess SC, Shiota M, Flakoll P, Donahue EP, Malloy CR, Sherry AD, Magnuson MA (2003). Mechanisms by which liver-specific PEPCK knockout mice preserve euglycemia during starvation. Diabetes.

[B11] Chirgwin JM, Prezybyla AE, MacDonald RJ, Rutter WJ (1979). Isolation of biologically active ribonucleic acid from sources enriched in ribonuclease. Biochemistry.

[B12] McGrane MM, deVente J, Yun J, Bloom J, Park EA, Wynshaw-Boris A, Wagner T, Rottman FM, Hanson RW (1988). Tissue-specific expression and dietary regulation of a chimeric PEPCK/bGH gene in transgenic mice. J Biol Chem.

[B13] Turnell DC, Cooper JD (1982). Rapid assay for amino acids in serum or urine by pre-column derivatization and reversed-phase liquid chromatography. Clin Chem.

[B14] Lo S, Russell JC, Taylor AW (1970). Analysis of glycogen in small samples. J Appl Physiol.

[B15] Garber AJ, Hanson RW (1971). The interelationships of varous pathways forming gluconeogenic precursors in guinea pig liver mitchondria.. J Biol Chem.

[B16] Gutmann I, Wahlefeld AW, Bergmeyer HU (1974). L-Malate: Determination with malate dehydrogenase and NAD. Methods of Enzymatic Analysis.

[B17] Gawehn K, Bergmeyer HU, Bergmeyer HU (1974). D-(-)-Lactate. Methods of Enzymatic Analysis.

[B18] Czok R, Lampecht W, Bergmeyer HU (1974). Pyruvate, phosphoenolpyruvate and D-glycrate-2-phosphate. Methods of Enzymatic Analysis.

[B19] Kun E, Kearney EB, Bergmeyer HU (1974). Ammonia. Methods of Enzymatic Analysis.

[B20] Ballard FJ, Hanson RW (1967). P-enolpyruvate carboxykinase and pyruvate carboxylase in the developing liver. Biochem J.

[B21] Burgess SC, Hausler N, Merritt M, Jeffrey FM, Storey C, Milde A, Koshy S, Lindner J, Magnuson MA, Malloy CR, Sherry AD (2004). Impaired tricarboxylic acid cycle activity in mouse livers lacking cytosolic phosphoenolpyruvate carboxykinase. J Biol Chem.

[B22] Vidnes J, Sovik O (1976). Gluconeogenesis in infancy and childhood III. Deficiency of the extramitochondrial form of hepatic phosphoenolpyruvate carboxykinase in a case of persistent neonatal hypoglycemia. Acta Pediatr Scand.

[B23] Zarzoli A, Turkenkopf IJ, Mueller VL (1969). Gluconeogenesis in developing rat kidney cortex. Biochem J.

[B24] Arinze IJ (1975). On the development of P-enolpyruvate carboxykinase and gluconeogenesis in guinea pig liver.. Biochem Biophys Res Comm.

[B25] Philippidis H, Ballard FJ (1969). The development of gluconeogenesis in rat liver: in vivo experiments. Biochem J.

[B26] Hanson RW, Mehlman MA (1976). Gluconeogenesis: Its role in mammalian species..

[B27] Hanson RW, Garber AJ (1972). P-enolpyruvate carboxykinase: I. Its role in gluconeogenesis. Amer J Clin Nutr.

[B28] Owen OE, Kalhan SC, Hanson RW (2002). The key role of anaplerosis and cataplerosis for citric acid cycle function. J Biol Chem.

[B29] Felig P, Owen OE, Wahren J, Cahill GFJ (1969). Amino acid metabolism during prolonged starvation. J Clin Invest.

[B30] Snell K, Duff DA (1977). The release of alanine by rat diaphragm muscle in vitro.. Biochem J.

[B31] Utter MF, Kurahashi K (1953). Mechanism of action of oxalacetate carboxylase from liver.. J Amer Chem Soc.

